# Glycerol Acrylate-Based Photopolymers with Antimicrobial and Shape-Memory Properties

**DOI:** 10.3390/polym16060862

**Published:** 2024-03-21

**Authors:** Evelina Saunoryte, Aukse Navaruckiene, Sigita Grauzeliene, Danguole Bridziuviene, Vita Raudoniene, Jolita Ostrauskaite

**Affiliations:** 1 Department of Polymer Chemistry and Technology, Kaunas University of Technology, Radvilenu Rd. 19, LT-50254 Kaunas, Lithuania; 2 Biodeterioration Research Laboratory, Nature Research Center, Akademijos Str. 2, LT-08412 Vilnius, Lithuania

**Keywords:** photocuring, glycerol acrylate, vanillin styrene, rheological properties, thermal properties, mechanical properties, shape-memory, antimicrobial activity

## Abstract

In this paper, for the first time, photopolymers were synthesized from glycerol acrylates with different numbers of functional groups, 2-hydroxy-3-phenoxypropyl acrylate, glycerol dimethacrylate or glycerol trimethacrylate, without and with the addition of vanillin styrene. The photocuring kinetics were monitored by real-time photorheometry. The mechanical, rheological, thermal, antimicrobial and shape-memory properties of the photopolymers were investigated. All polymers synthesized demonstrated antibacterial activity against *Escherichia coli* and *Staphylococcus aureus,* as well as antifungal activity against *Aspergillus flavus* and *Aspergillus niger*. 2-Hydroxy-3-phenoxypropyl acrylate-based polymers showed thermoresponsive shape-memory behavior. They were able to maintain their temporary shape below the glass transition temperature and return to their permanent shape above the glass transition temperature. Synthesized photopolymers have potential to be used as sustainable polymers in a wide range of applications such as biomedicine, photonics, electronics, robotics, etc.

## 1. Introduction

Biobased materials are a viable alternative to petroleum-based materials, as they are environmentally friendly and come from biorenewable sources, such as plants and living systems [1,2]. These materials can be chemically modified by the addition of functional groups to produce monomers suitable for polymerization [3]. A biobased material with great potential for polymer synthesis is glycerol, which is obtained as the main by-product of the biodiesel production process [4]. Glycerol is widely used in the polymer industry as a feedstock for polymer synthesis [5]. Another example of promising materials is vanillin [6]. It is a biobased aromatic compound that can be extracted directly from plants or obtained through the chemical modification of lignin [7]. Vanillin-based materials have been successfully used in polymer synthesis and have shown high mechanical strength and good antimicrobial properties [8]. Both vanillin and glycerol derivatives were used successfully in the photopolymerization of biobased polymers [9,10].

Photopolymerization is an environmentally friendly polymerization process that follows the principles of Green Chemistry [11]. It is a fast process that starts rapidly after irradiation, and polymers can be produced in a few minutes, which is a huge advantage compared to thermal polymerization [12]. Moreover, photopolymerization is an energy-efficient process and can be performed at room temperature [13]. It is an easily controlled process, since it only starts in irradiated areas, which makes photopolymerization suitable for optical 3D printing technology [14]. In addition, photopolymerization can be used as a simple way to obtain polymers with unique properties such as antimicrobial activity or shape-memory [15].

Shape-memory materials are smart materials that can change their shape when triggered by a stimulus [16]. The wide range of stimuli, such as light, magnetic, thermal, chemical or electrical [17], allows for the use of shape-memory polymers in the production of smart materials [18]. Thermoresponsive shape-memory polymers obtain temporary shapes when they are deformed at a temperature higher than their glass transition temperature, and then, cooled below it again [19]. They can be used in various application areas such as biomedicine, photonics, electronics, robotics, etc. [20].

Antimicrobial materials are very important nowadays as they can prevent the spread of diseases [21]. There are two main types of antimicrobial materials: natural and synthetic [22]. Synthetic antimicrobial polymers are mostly obtained through the incorporation of biocidic organic compounds such as essential oils or biocidic inorganic particles such as metal ions into the polymeric material. The main disadvantage of these approaches is that their antimicrobial activity decreases over time [23]. Due to this, the most advantageous approach is the chemical modification of polymers, i.e., the covalent incorporation of antimicrobial fragments [24]. The main application areas for antimicrobial polymers are the medicine, textile and food industries [25]. For example, the use of antimicrobial polymers in the manufacture of medical devices could stop most hospital-acquired infections, which occur because medical devices provide the right conditions for microbes to grow [26]. Antimicrobial food packaging could stop disease transmission through food and even prevent food from spoiling during storage [27].

In this study, three glycerol derivatives, 2-hydroxy-3-phenoxypropyl acrylate, glycerol dimethacrylate and glycerol trimethacrylate, were used in free-radical photopolymerization without and with the addition of vanillin styrene, which was used to improve the mechanical properties of the resulting polymers. A total of 3 mol.% of photoinitiator diphenyl(2,4,6-trimethylbenzoyl)phosphine oxide was used in the compositions. This photoinitiator was selected due to its photobleaching effect and its ability to cure deep layers of resin [28]. The aim of this study was to develop antimicrobial thermoresponsive shape-memory photopolymers. For the first time, vanillin styrene was used in photopolymerization. It was determined that the addition of vanillin styrene increased the rigidity and thermal stability of the polymer prepared with 2-hydroxy-3-phenoxypropyl acrylate, but also, the bulk structure of this substituent increased the free volume in the polymer network, which could have contributed to the slightly inferior antimicrobial properties of the polymer films.

## 2. Materials and Methods

### 2.1. Materials

**Figure 1:**
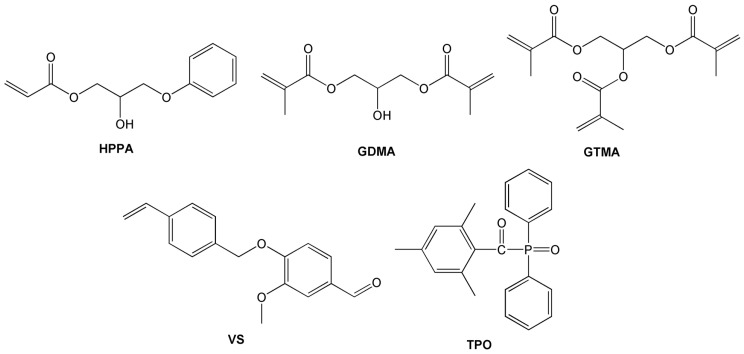
Chemical structures of 2-hydroxy-3-phenoxypropyl acrylate (HPPA), glycerol dimethacrylate (GDMA), glycerol trimethacrylate (GTMA), vanillin styrene (VS) and diphenyl(2,4,6-trimethylbenzoyl)phosphine oxide (TPO).

2-hydroxy-3-phenoxypropyl acrylate (HPPA, Sigma-Aldrich, Saint Louis, MO, USA), glycerol dimethacrylate (GDMA, Sigma-Aldrich, Saint Louis, MO, United States), glycerol trimethacrylate (GTMA, “abcr” GmbH, Karlsruhe, Germany), vanillin styrene (VS, Specific Polymers, Castries, France) and diphenyl(2,4,6-trimethylbenzoyl)phosphine oxide (TPO, Fluorochem, Glossop, UK) (Figure 1) were used as received.

### 2.2. Preparation of Polymer Specimens

The initial mixtures containing 1 mol of the glycerol derivative (HPPA, GDMA or GTMA), 0.1 mol of VS (in **B1**–**B3** cases) and 3 mol.% of TPO were stirred with a magnetic stirrer at 40 °C for 10 min until a homogenous mixture was obtained. The mixtures were then poured into Teflon molds (45 mm × 10 mm × 3 mm) and cured for 1–3 min (until the samples had hardened) under a UV/Vis lamp (Helios Italquartz, model GR.E 500 W, Milan, Italy) with an intensity of 310 mW/cm^2^. The compositions of the resins are presented in Table 1.

### 2.3. Characterization Techniques

A Spectrum BX II FT-IR spectrometer (Perkin Elmer, Llantrisant, UK) was used to record Fourier transform infrared spectroscopy (FT-IR) spectra. The reflection was measured during the test. The range of wavenumbers was (4000–650) cm^−1^.

Polymer **B1** FT-IR (cm^−1^): 3467 (ν, -OH); 3062 (ν, C-H aromatic); 2929 (ν, C-H aliphatic); 1717 (ν, C=O); 1637 (ν, C=C); 1156 (ν, C-C).

Polymer **B2** FT-IR (cm^−1^): 3452 (ν, -OH); 3075 (ν, C-H aromatic); 2933 (ν, C-H aliphatic); 1723 (ν, C=O); 1636 (ν, C=C); 1159 (ν, C-C).

Polymer **B3** FT-IR (cm^−1^): 3106 (ν, C-H aromatic); 2958 (ν, C-H aliphatic); 1721 (ν, C=O); 1637 (ν, C=C); 1153 (ν, C-C).

Polymer **B4** FT-IR (cm^−1^): 3451 (ν, -OH); 3062 (ν, C-H aromatic); 2981 (ν, C-H aliphatic); 1719 (ν, C=O); 1638 (ν, C=C); 1158 (ν, C-C).

Polymer **B5** FT-IR (cm^−1^): 3438 (ν, -OH); 2925 (ν, C-H aliphatic); 1721 (ν, C=O); 1637 (ν, C=C); 1161 (ν, C-C).

Polymer **B6** FT-IR (cm^−1^): 2983 (ν, C-H aliphatic); 1720 (ν, C=O); 1637 (ν, C=C); 1151 (ν, C-C).

Soxhlet extraction was used to determine the yield of insoluble fractions. Polymer samples of 0.4 g were extracted with acetone for 24 h. A swelling test was also performed using two different solvents, acetone and toluene. The methodology of these experiments was described in a previous publication [29].

Thermal characteristics of the polymers were investigated using thermogravimetric analysis (TGA) and dynamic mechanical thermal analysis (DMTA). During DMTA, the shear mode was used with a frequency of 1 Hz, a shear strain of 0.1% and a normal force of 5 N. The mechanical characteristics of the synthesized polymers were determined by a tensile test. Young’s modulus, tensile strength and elongation at break were determined. The methodology of these experiments was described in a previous publication [29].

The collected data were statistically analyzed using ANOVA for the Microsoft Excel program. The results are presented as the average values ± standard deviation. The estimated *p*-value was below 0.05 within the groups.

### 2.4. Real-Time Photorheometry

UV/Vis curing tests were performed with glycerol-based resins containing 0.1 mol of VS (only in **B1**–**B3** cases) and 3 mol.% of TPO on a MCR302 rheometer (Anton Paar, Graz, Austria) equipped with a plate/plate measuring system using the methodology described in a previous publication [29]. In all cases, shear mode with a frequency of 10 Hz and a shear strain of 0.1% was used. Five measurements were made for each formulation, and results with a standard deviation of less than 5% were obtained.

### 2.5. Antimicrobial Testing

The antimicrobial testing of polymers was performed using the methodology described in a previous publication [29]. The final inoculum concentrations were 5.5 × 10^8^ colony-forming units/mL (CFU/mL) for *Escherichia coli* (*E. coli*), 7.0 × 10^8^ for *Staphylococcus aureus* (*S. aureus*), 1.0 × 10^7^ for *Aspergillus niger* (*A. niger*) and 1.7 × 10^7^ for *Aspergillus flavus* (*A. flavus*).

## 3. Results and Discussion

### 3.1. Monitoring of Photocuring Kinetics by Real-Time Photorheometry

Real-time photorheometry was used to investigate the photocuring kinetics of the resins. The results are summarized in Table 2.

**Figure 2:**
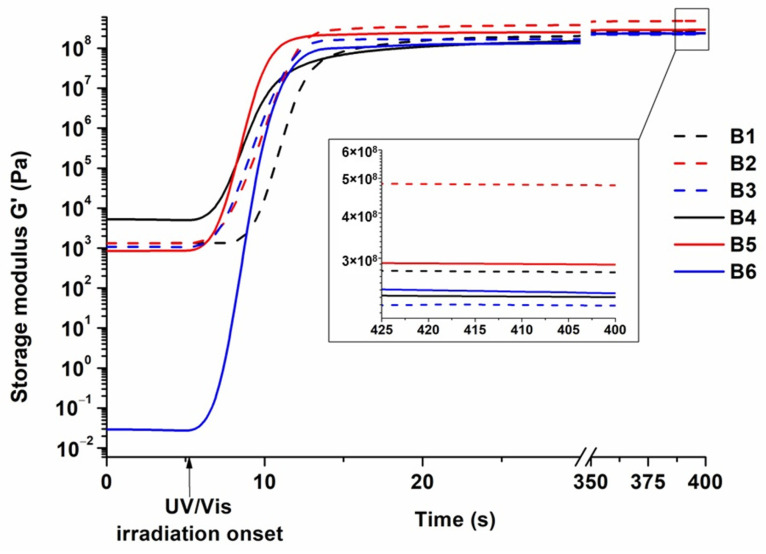
Dependence of storage modulus of resins **B1**–**B6** on irradiation time.

The storage modulus represents the rigidity of the polymers. The dependences of the storage modulus of resins **B1–B6** on irradiation time are shown in Figure 2. More rigid polymers were obtained from compositions with VS (**B1** and **B2**) compared to compositions without VS (**B4** and **B5**) due to the two rigid benzene rings in the VS structure. The only exceptions were the polymers prepared with GTMA, as the value of G’ of polymer **B3** prepared with VS was slightly lower than that of polymer **B6**. The reason for this might be the high number of functional groups of GTMA, in which molecules tend to react with each other instead of VS.

Photocuring (determined by gel point) was faster when VS was not used in the compositions, because acryl groups are more reactive than vinyl groups [30]. However, the photocuring of GTMA-based polymer **B3** with VS was slightly faster (t_gel_ = 2.4 s) than that of GTMA-based polymer **B6** (t_gel_ = 2.6 s). This can be explained by the higher number of functional groups present in compositions with GTMA and faster formation of spatial hindrances that slow down photopolymerization, because the remaining groups cannot reach each other [31].

Polymer shrinkage was in the range of 2–14%. Higher shrinkage values were observed for compositions with VS (**B2** and **B3**); the only exceptions were compositions **B1** and **B4** prepared with HPPA. Polymers **B2** and **B3** shrunk more than polymers **B5** and **B6** due to the free volume in the polymer network. Also, an increased number of functional groups increases the shrinkage of resultant polymers due to the increased formation of short covalent bonds [32]. A high number of functional groups results in a more rigid polymer network, which lowers the volume of the resulting polymer. Compositions **B1** and **B4** formed a linear polymer, which shrank less than the cross-linked polymers **B2**, **B3**, **B5** and **B6** [33]. The rate of photocuring also influenced the shrinkage of polymers, and the gel point value of polymer **B1** was higher than that of polymer **B4**, indicating slower photocuring and less shrinkage [34].

### 3.2. Characterization of Polymer Structure

**Figure 3:**
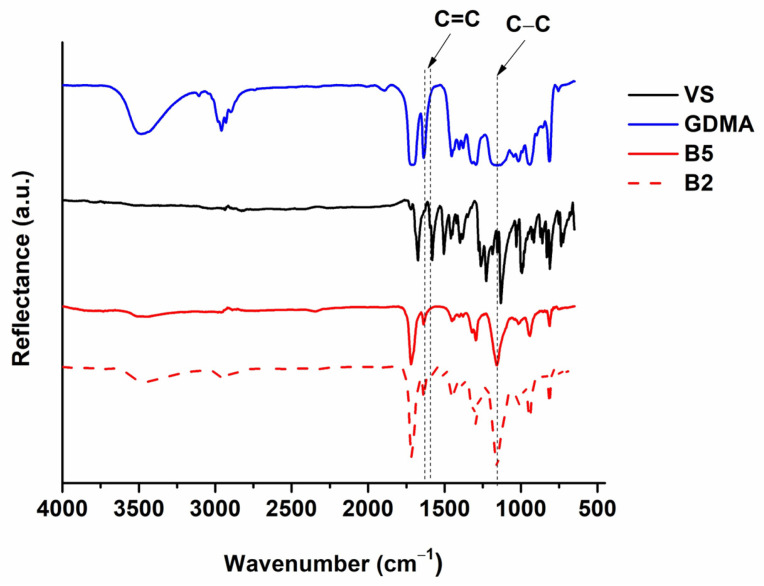
FT-IR spectra of GDMA, VS and polymers **B2** and **B5**.

The chemical structure of the polymers was confirmed by FT-IR spectroscopy. The FT-IR spectra of the polymers were compared with those of the monomers in order to observe the decrease in the signals of the functional C=C groups and the appearance of new signals as a result of the formation of C-C bonds. As an example, the FT-IR spectra of GDMA, VS and polymers **B2** and **B5** are presented in Figure 3. The signals of the acryl groups at 1637 cm^−1^ and vinyl groups at 1581 cm^−1^ are reduced in the polymer spectra. In addition, a newly formed C-C bond signal is visible at 1156 cm^−1^ in the spectra of the polymers. These changes indicate that polymers were formed during photocuring.

To confirm the formed structure of the polymers, the yield of the insoluble fraction was calculated after Soxhlet extraction and swelling tests were performed. The results are presented in Table 3. The yield of the insoluble fraction of the polymers was in the range of 86–98%. The highest value was shown by GTMA- and VS-based polymer **B3** (98%) due to the densely cross-linked network. The lowest value was shown by an HPPA-based polymer without VS (**B4**). The reason for this is the physical network of the linear macromolecules, which is formed through the steric entanglement of large aromatic substituents, and the resulting steric constraints prevent the linear macromolecules from entangling while dissolving in the solvent. In all cases, the higher amount of yield of the insoluble fraction was obtained for polymers prepared with VS compared to the corresponding polymers without VS.

**Figure 4:**
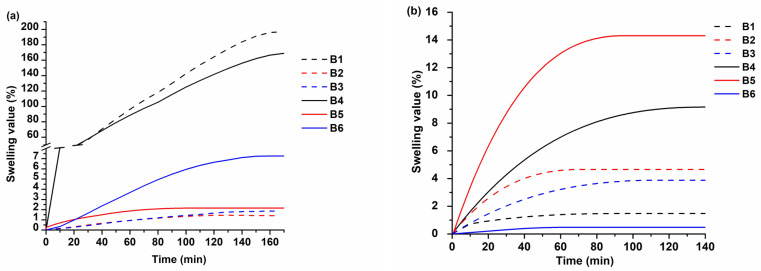
Dependence of swelling value on the duration of swelling of polymers **B1–B6** in acetone (**a**) and toluene (**b**).

Two solvents, the polar solvent acetone and the non-polar solvent toluene, were selected for the swelling test (Table 3, Figure 4). The highest swelling values were shown by HPPA-based polymers **B1** and **B4** in acetone. The reason for this is the low dense physical network of these polymers, caused by linear macromolecules and bulk aromatic VS substituents, and their solubility in acetone. However, their swelling values in toluene were lower (1.48–9.14%) indicating that these polymers are not easily soluble in toluene. GDMA- and GTMA-based polymers (**B2**, **B3**, **B5** and **B6**) showed higher swelling values in toluene than in acetone due to their structure being more similar to the non-polar solvent toluene than to the polar solvent acetone [35].

### 3.3. Thermal Properties

**Figure 5:**
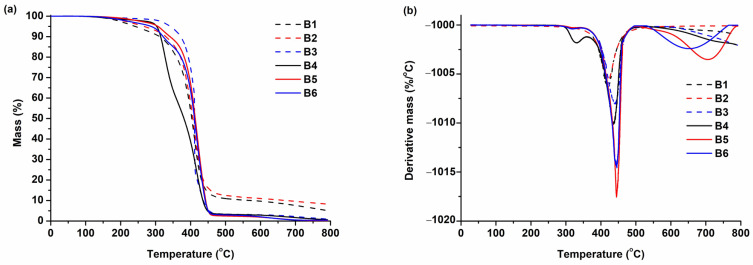
Thermogravimetric curves (**a**) and differential thermogravimetric curves (**b**) of polymers **B1–B6**.

Thermogravimetric analysis and dynamic mechanical thermal analysis were selected to determine the thermal characteristics of the synthesized polymers. The results are presented in Table 4. The thermal decomposition of polymers occurred in one or two steps, depending on the polymer (Figure 5). The thermal decomposition of polymers **B1**–**B3** (prepared with VS) occurred in one step, while the thermal decomposition of polymers **B4**–**B6** (prepared without VS) occurred in two steps. The reason for this was the presence of a higher amount of low-molecular-weight compounds and/or oligomers that were not incorporated into the polymer network of polymers **B4**–**B6**, which thermally decompose easier than the cross-linked parts of these polymers. The highest thermal stability was demonstrated by polymer **B3** (T_dec-10%_ = 372 °C). This polymer had the highest yield-of-insoluble-fraction value, which makes it thermally stable at higher temperatures. Polymers **B1** and **B4** composed of linear macromolecules had a temperature of 10% weight loss in the range of 312–314 °C. All polymers demonstrated higher thermal stability compared to the GDMA- and vanillin acrylate-based polymers used for additive manufacturing (T_dec-10%_ = 250–280 °C) [36].

**Figure 6:**
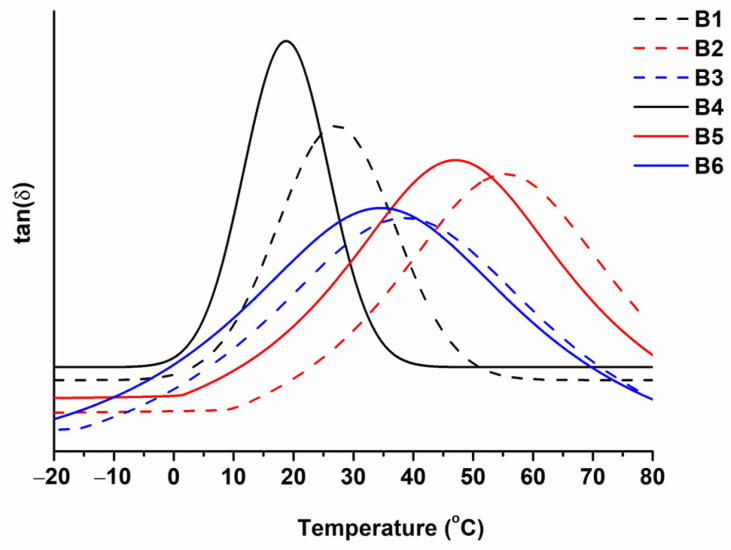
DMTA curves of polymers **B1–B6**.

The glass transition temperature of the synthesized polymers was in the range of 19–55 °C. The DMTA curves are presented in Figure 6. Polymers **B1**–**B3** prepared with VS were determined to have a higher glass transition temperature than the corresponding polymers prepared without VS (**B4**–**B6**). This was because of the aromatic fragments of VS in their structure. Polymers **B1** and **B4** showed the lowest T_g_ values due to their linear macromolecules. The highest values of T_g_ were demonstrated by polymers **B2** and **B5** prepared with GDMK. These polymers have a cross-linked structure and the highest values of insoluble fraction, which results in higher values of T_g_.

### 3.4. Shape-Memory Properties of Polymers

**Figure 7:**
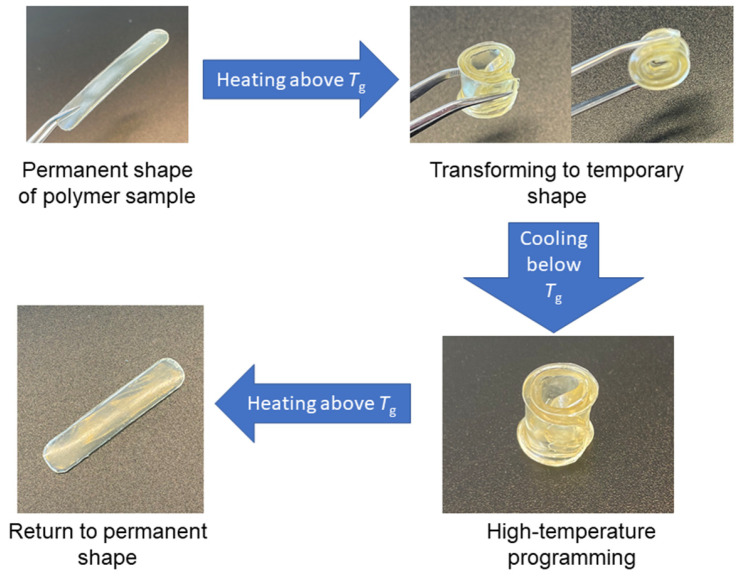
Shape-memory behavior of polymer **B1**.

Polymers **B1** and **B4** demonstrated thermo-responsive shape-memory properties, which were determined by T_g_ [37]. These polymers were composed of macromolecules with hard and soft fragments. Soft aliphatic chains act as switchable units that allow the polymer to change its shape [38]. These polymer samples were able to obtain a temporary shape and maintain it when the temperature was lower than their T_g_. To demonstrate these properties, the polymer samples were heated above their glass transition temperature, and then, deformed to the desired temporary shape, in this case, a roll. When the temporary shapes were obtained, they were fixed by cooling the polymer samples below their T_g_. The temporary shapes did not change over time while the samples were kept at a temperature below their T_g_. The ability of the samples to return to their permanent shape was verified by heating them above their T_g_. Polymers **B1** and **B4** were able to return to their permanent shapes in a short period of time, 5 s. The heating–cooling–heating cycle scheme of the polymer **B1** sample is presented in Figure 7.

### 3.5. Mechanical Characteristics

A tensile test was performed to determine the mechanical characteristics of polymers **B1**–**B6**. The results are presented in Table 5. Polymers **B1** and **B4** prepared with HPPA were soft and flexible and showed high values of elongation at break and low Young’s modulus values due to the linear macromolecule structure. The most rigid polymers and the highest Young’s modulus values were obtained for polymers **B2** and **B5** prepared with GDMA. These polymers have the highest yield-of-insoluble-fraction values. Because of this, these polymers are mechanically stronger than the linear polymers **B1** and **B4**, but their elongation-at-break values are lower. Polymers prepared with GDMA showed similar Young’s modulus values to antimicrobial GDMA-based polymers suitable for optical 3D printing (Young’s modulus = 76.6–190.7 MPa) [39]. Higher values of Young’s modulus and lower values of tensile strength were shown by polymers **B4**–**B6** prepared without VS compared to the corresponding polymers **B1**–**B3** prepared with VS. The only exceptions are polymers **B1** and **B4** prepared with HPPA. In this case, higher values of Young’s modulus were shown by polymer **B1** prepared with VS. The reason for this is the presence of aromatic rings in the structure of VS, which increase the rigidity of the linear polymer **B1** prepared with HPPA. However, the addition of VS to GDMA or GTMA had the opposite effect due to the increased the free volume in the polymer network, as it created a slightly more flexible polymer network, resulting in a lower Young’s modulus and a slightly higher value of elongation at break.

### 3.6. Antimicrobial Activity of Polymers

The antibacterial and antifungal activity of the polymer films was investigated during direct contact of the bacterial or fungal suspensions with the polymer samples and by calculating the percent reduction of microbial spores after 1 h to see their dependence on the polymer structure. The results are presented in Table 6.

All polymers were more active against the Gram-negative bacteria *E. coli* compared to the Gram-positive bacteria *St. aureus*. This is because *St. aureus* is more resistant to antibiotics and antimicrobial agents than *E. coli* [40]. For example, the highest antibacterial activity was demonstrated by polymer **B6**, with a 99.12% reduction against *E. coli* and an 88.38% reduction against *St. aureus*. All polymers prepared with VS were less active against both types of bacteria compared to the corresponding polymers prepared without VS. The structure of VS increased the free volume and facilitated molecular motions, which could impact the decreased antibacterial properties of the polymer films. For example, the VS-based polymer **B2** reduced the viability of *St. aureus* by 72.95%, while the corresponding polymer without VS (**B5**) reduced it by 78.09%. The reason for this is the high antimicrobial activity of glycerol derivatives [41]. The lowest antibacterial activity was shown by polymers **B1** and **B4** prepared with HPPA, and the highest by polymers **B3** and **B6** prepared with GTMA. For example, HPPA-based polymer **B4** reduced the viability of *E. coli* by 82.30%, while GTMA-based polymer **B6** reduced it by 99.12%. The reason for this is the higher number of antibacterial carbonyl groups in GTMA compared to HPPA [42].

The antifungal activity of the polymer films was tested against two microscopic fungi, *A. flavus* and *A. niger*. In all cases, the activity of polymers against both fungi was affected by the concentration of carbonyl groups and increased with an increase in their concentration [38]. However, all polymers demonstrated very similar activity against *A. flavus* (94.04–96.85%) and *A. niger* (89.97–92.50%), with less than a 3% difference compared to other vanillin-based polymers, with 93.1–100.0% activity against *A. flavus* and 72.3–92.5% activity against *A. niger* [29].

## 4. Conclusions

We synthesized photocurable resins composed of 2-hydroxy-3-phenoxypropyl acrylate, glycerol dimethacrylate or glycerol trimethacrylate with and without vanillin styrene. This study comprehensively characterized the synthesized polymers, focusing on their photocuring kinetics, structure, thermal properties, shape-memory behavior, mechanical characteristics and antimicrobial activity. To summarize the findings of this study, three key conclusions may be drawn:

- Enhanced polymer properties with vanillin styrene: The addition of vanillin styrene in the photopolymer formulation led to improved rigidity and thermal stability. However, this enhancement came at the cost of reduced antibacterial activity. This suggests that when designing polymers, mechanical properties and antibacterial activity must be balanced.
- High antimicrobial activity: All synthesized polymers demonstrated high antibacterial activity against *Escherichia coli* (71.78–99.12%) and *Staphylococcus aureus* (54.68–88.38%), as well as antifungal activity against *Aspergillus flavus* (94.04–96.85%) and *Aspergillus niger* (89.97–92.50%). Even after just one hour of contact, the polymers exhibited significant antimicrobial activity, suggesting their potential benefit in various antimicrobial applications.
- Thermoresponsive shape-memory properties: Polymers based on 2-hydroxy-3-phenoxypropyl acrylate showed thermoresponsive shape-memory behavior. They were able to keep their temporary shape below the glass transition temperature while also returning to their permanent shape above it.

This comprehensive characterization offers valuable insights into the tailoring of polymer properties for diverse applications, highlighting avenues for further optimization and exploration. The obtained new biobased photopolymers with thermoresponsive shape-memory properties could be applied to optical 3D printing, biomedical devices, electronics, etc.

## Figures and Tables

**Table 1:** Compositions of resins **B1**–**B6**.

Resin	Glycerol Derivative	Molar Ratio of Glycerol Derivative and Vanillin Styrene	Amount of Photoinitiator TPO, mol.% *
**B1**	HPPA	1:0.1	3
**B2**	GDMA	1:0.1	3
**B3**	GTMA	1:0.1	3
**B4**	HPPA	1:0	3
**B5**	GDMA	1:0	3
**B6**	GTMA	1:0	3

* Calculated from the total mol amount of monomers.

**Table 2:** Mechanical and rheological characteristics of the resins.

Resin	Storage Modulus G′ *, MPa	Loss Modulus G″ *, MPa	Complex Viscosity *, mPa·s	Gel Point, t_gel_, s	Shrinkage, %
**B1**	281 ± 4	97.8 ± 2.0	4.73 ± 0.09	1.8 ± 0.0	2 ± 0.0
**B2**	489 ± 7	98.8 ± 1.6	7.94 ± 0.12	2.3 ± 0.1	14 ± 0.5
**B3**	224 ± 8	52.5 ± 0.8	3.66 ± 0.06	2.4 ± 0.0	13 ± 0.4
**B4**	238 ± 6	199.0 ± 2.1	4.93 ± 0.04	1.3 ± 0.1	5 ± 0.2
**B5**	293 ± 5	33.3 ± 0.4	4.69 ± 0.05	0.3 ± 0.0	11 ± 0.4
**B6**	251 ± 4	121.0 ± 1.5	4.44 ± 0.04	2.6 ± 0.1	8 ± 0.0

* After 450 s from the start of irradiation.

**Table 3:** Characteristics of polymers.

Polymer	Yield of Insoluble Fraction, %	Swelling Values in Acetone, %	Swelling Values in Toluene, %
**B1**	93 ± 0.2	197.2 ± 3.2	1.48 ± 0.1
**B2**	94 ± 0.1	1.4 ± 0.1	4.66 ± 0.2
**B3**	98 ± 0.5	1.9 ± 0.1	3.88 ± 0.1
**B4**	86 ± 0.3	169.3 ± 2.0	9.14 ± 0.2
**B5**	94 ± 0.1	2.2 ± 0.1	14.31 ± 0.3
**B6**	89 ± 0.2	7.3 ± 0.2	0.48 ± 0.0

**Table 4:** Thermal characteristics of polymers.

Polymer	T_dec-10%_, °C *	T_g_, °C **
**B1**	312	27
**B2**	329	55
**B3**	372	39
**B4**	314	19
**B5**	341	47
**B6**	321	35

* From TGA curves. ** From DMTA curves.

**Table 5:** Mechanical characteristics of polymers obtained by tensile test.

Polymer	Young’s Modulus, MPa	Tensile Strength, MPa	Elongation at Break, %
**B1**	46.6 ± 0.9	1.9 ± 0.1	139.0 ± 5.2
**B2**	167.2 ± 1.2	2.1 ± 0.1	1.9 ± 0.0
**B3**	73.5 ± 1.0	0.5 ± 0.0	3.3 ± 0.1
**B4**	6.4 ± 0.0	0.5 ± 0.0	291.4 ± 7.6
**B5**	219.9 ± 3.5	1.9 ± 0.1	1.7 ± 0.0
**B6**	96.9 ± 1.1	0.3 ± 0.0	2.9 ± 0.1

**Table 6:** Antimicrobial characteristics of polymers.

Polymer	Reduction in Microbial Spores (CFU/mL) after 1 h, %			
	*Escherichia coli*	*Staphylococcus aureus*	*Aspergillus flavus*	*Aspergillus niger*
**B1**	71.78 ± 0.20	64.68 ± 0.35	94.04 ± 0.10	89.97 ± 0.15
**B2**	73.51 ± 0.35	72.95 ± 0.40	95.13 ± 0.20	90.84 ± 0.10
**B3**	78.18 ± 0.20	74.81 ± 0.20	95.77 ± 0.25	91.27 ± 0.10
**B4**	82.30 ± 0.45	75.14 ± 0.25	95.80 ± 0.10	91.72 ± 0.05
**B5**	87.82 ± 0.50	78.09 ± 0.30	96.63 ± 0.10	91.93 ± 0.05
**B6**	99.12 ± 0.20	88.38 ± 0.10	96.85 ± 0.10	92.50 ± 0.10

## Data Availability

Data are available on request.
